# Microbiota-Gut-Brain Communication in the SARS-CoV-2 Infection

**DOI:** 10.3390/cells10081993

**Published:** 2021-08-06

**Authors:** Luana M. Manosso, Camila O. Arent, Laura A. Borba, Luciane B. Ceretta, João Quevedo, Gislaine Z. Réus

**Affiliations:** 1 Translational Psychiatry Laboratory, Graduate Program in Health Sciences, University of Southern Santa Catarina (UNESC), Criciúma 77054-000, SC, Brazil; psiquiatria@unesc.net (L.M.M.); camilaarent@unesc.net (C.O.A.); lauraborba28@gmail.com (L.A.B.); Joao.L.DeQuevedo@uth.tmc.edu (J.Q.); 2 Programa de Pós-Graduação em Saúde Coletiva, Universidade do Extremo Sul Catarinense, Criciúma 88806-000, SC, Brazil; ppgscol@unesc.net; 3 Translational Psychiatry Program, Department of Psychiatry and Behavioral Sciences, McGovern Medical School, The University of Texas Health Science Center at Houston (UTHealth), Houston, TX 77030, USA; 4 Center of Excellence on Mood Disorders, Department of Psychiatry and Behavioral Sciences, McGovern Medical School, The University of Texas Health Science Center at Houston (UTHealth), Houston, TX 77030, USA; 5 Neuroscience Graduate Program, The University of Texas Graduate School of Biomedical Sciences at Houston, Houston, TX 77030, USA

**Keywords:** microbiota-gut-brain axis, neuroinflammation, SARS-CoV-2, mood disorders, neurological diseases, COVID-19

## Abstract

The coronavirus disease of 2019 (COVID-19) is an infectious disease caused by severe acute respiratory syndrome 2 (SARS-CoV-2). In addition to pneumonia, individuals affected by the disease have neurological symptoms. Indeed, SARS-CoV-2 has a neuroinvasive capacity. It is known that the infection caused by SARS-CoV-2 leads to a cytokine storm. An exacerbated inflammatory state can lead to the blood–brain barrier (BBB) damage as well as to intestinal dysbiosis. These changes, in turn, are associated with microglial activation and reactivity of astrocytes that can promote the degeneration of neurons and be associated with the development of psychiatric disorders and neurodegenerative diseases. Studies also have been shown that SARS-CoV-2 alters the composition and functional activity of the gut microbiota. The microbiota-gut-brain axis provides a bidirectional homeostatic communication pathway. Thus, this review focuses on studies that show the relationship between inflammation and the gut microbiota–brain axis in SARS-CoV-2 infection.

## 1. SARS-CoV-2

In December 2019, a pneumonia outbreak was reported in Wuhan, China. This disease, now named coronavirus disease 2019 (COVID-19), is an infectious disease caused by severe acute respiratory syndrome 2 (SARS-CoV-2). The COVID-19 quickly spread across the continent, and in March of 2020, the World Health Organization (WHO) declared COVID-19 to be a pandemic [1]. Noteworthy, this pandemic is devastating public health and economies, with the sum of more than 175 million laboratory-confirmed COVID-19 cases infected and 3.7 million dead to date [2].

The angiotensin-converting enzyme 2 (ACE2) has been identified as the primary target of the SARS-CoV-2 for cell surface attachment and likely entry into the host cell. The coronavirus spike protein (a transmembrane fusion protein found in the virion envelope) facilitates entry of the virus into target cells through the engaged ACE2 as the entry receptor. Moreover, cell entry requires priming of the spike protein by the cellular serine protease TMPRSS2 or other proteases [3,4]. Furthermore, an interaction between host cell receptor CD147 and SARS-CoV-2 spike protein can be another route to facilitate the infection [5,6]. 

The immune response is a determinant in the pathogenesis of COVID-19 [4]. The release of large amounts of pro-inflammatory cytokines, in an event known as “cytokine storm” [7,8], can converge on changes to the target tissues and host physiology and can be associated with the severity of COVID-19 [9]. 

The clinical manifestations in cases of COVID-19 vary widely, from asymptomatic cases to cases with septic shock and multiple organ failure [10]. The most affected system by COVID-19 is the pulmonary system, with the most frequent clinical manifestations including cough, fever, dyspnea, and sore throat. In severe cases, pneumonia, acute hypoxic respiratory failure, and/or death can occur [11]. However, extrapulmonary organs and systems are also affected by COVID-19, which could have significant health consequences. Frequent symptoms include hematologic, cardiovascular, renal, hepatobiliary, endocrinologic, dermatologic, ophthalmologic, gastrointestinal, and neurologic and neuropsychiatric manifestations [4,11], including headache and dizziness [4,12,13], anxiety, depression [14,15], and cognitive decline [16]. It is noteworthy that, although several factors may be involved, the increase in inflammatory cytokines can have a significant impact on these neurological symptoms [17]. Additionally, the temporal correlation between neurological and gastrointestinal symptoms in COVID-19 patients suggests that the microbiota-gut-brain axis can also be involved [18,19].

Considering the points raised, the objective of this review is to discuss the relationship of COVID-19 with mood disorders and neurodegenerative diseases, highlighting the role of inflammation and the microbiota-gut-brain axis. For that, we approach general aspects of mood disorders and neurodegenerative diseases. Next, we detail the role of inflammation and the microbiota-gut-brain axis. Afterward, we discuss the effects of SARS-CoV-2 on systemic inflammation and neuroinflammation. Finally, we bring evidence of the SARS-CoV-2 relationship in the microbiota-gut-brain axis.

## 2. General Aspects of Psychiatric Disorders and Neurodegenerative Diseases

Psychiatric disorders, especially mood disorders, have a high prevalence and compromise the well-being of those affected [20]. Major depressive disorder (MDD) is a mood disorder that is a leading cause of disability worldwide [21]. MDD is a multifactorial and polygenic disorder. The pathophysiology of MDD is not fully understood, but an overlap of multiple factors are involved, including decreased monoaminergic neurotransmitters (serotonin, dopamine, and norepinephrine), deregulation in the hypothalamic–pituitary–adrenal (HPA) axis, decreased neurotrophic factors, particularly brain-derived neurotrophic factor (BDNF), increased in glutamate (the major excitatory neurotransmitter in the central nervous system (CNS)), and increased inflammation and oxidative stress [22,23,24,25].

Neurodegenerative diseases such as Alzheimer’s and Parkinson’s diseases are age-associated conditions with progressive loss of specific neuronal structure and function. Alzheimer’s disease is characterized by cognitive impairment and Parkinson’s disease by a cardinal motor feature (rigidity or rest tremor). However, both neurodegenerative diseases are also associated with neuropsychiatric symptoms [26,27]. Furthermore, these neurodegenerative diseases are associated with protein misfolding and aggregation of amyloid-β peptide (in Alzheimer’s disease) and α-synuclein (in Parkinson’s disease). Moreover, other features of these diseases are oxidative stress, inflammation, and increased glutamate [28,29,30,31,32]. Alzheimer’s disease is also associated with decreased acetylcholine [33] and Parkinson’s disease with decreased dopamine [27].

## 3. Inflammation, Psychiatric Disorders, and Neurodegenerative Diseases

An adequate immune response and inflammation are essential for life. However, a non-resolving inflammation or a systemic chronic inflammation can be involved in several diseases [34,35], including cardiovascular disease [36], type 2 diabetes mellitus [37], cancer [38], psychiatric disorders [39], and neurodegenerative disorders [40].

Several meta-analyses highlighted an increase in systemic inflammatory markers in depressed individuals as compared with controls, especially interleukin-6 (IL-6), tumor necrosis factor-alpha (TNF-α), and C-reactive protein (CRP) [41,42,43,44,45]. In addition, neuroinflammation is also present in individuals with MDD. Indeed, a systematic review and meta-analysis of studies examining cerebrospinal fluid showed an increase in IL-6 and TNF-α in patients with MDD [46].

Inflammation also impacts neurodegenerative diseases [30,31]. A meta-analysis demonstrated that Alzheimer’s disease individuals had higher peripheral inflammatory markers than compared healthy controls. Additionally, IL-6 was associated with the severity of cognitive impairment [47]. Another meta-analysis showed that Parkinson’s disease individuals have an increase in peripheral cytokine levels (including inflammatory cytokine such as IL-6, TNF-α, CRP, IL-1β, and IL-2, and anti-inflammatory cytokine IL-10) [48]. Moreover, another meta-analysis also highlighted an increase in the inflammatory cytokines in the cerebrospinal fluid of patients with Alzheimer’s disease and Parkinson’s disease [49].

It is well established that peripheral cytokines can reach the CNS and contribute to neuroinflammation through cellular, humoral, and neural pathways [50,51]. Here, it is worth highlighting the role of the blood–brain barrier (BBB) integrity. Peripheral cytokines can increase BBB permeability or be transported through the circumventricular organs (regions where the BBB is less restrictive) [50,51,52]. Interestingly, BBB dysfunction can contribute to the onset and/or progression of psychiatric disorders [53,54] and neurodegenerative diseases [55,56].

It is worth noting that several conditions throughout life can contribute to peripheral inflammation, highlighting stress, smoking, physical inactivity, obesity, unhealthy diet, altered gut microbiota, and gut permeability [57,58]. Of note, a continually emerging body of evidence supports the role of the gut microbiota in systemic inflammation [59,60], psychiatric disorder, and neurodegenerative disorders [58,61,62,63].

## 4. Microbiota-Gut-Brain Axis, Psychiatric Disorders, and Neurodegenerative Diseases

The microbiota-gut-brain axis provides a bidirectional communication pathway between the brain and gastrointestinal tract [61,64,65]. The human microbiota is defined as the assemblage of living microorganisms present in the human body. Of note, the gut contains over 70% of all the microbes in the human body [66]. It is worth mentioning that the microbiota composition is host-specific and can change according to age, sex, medication, diet, genetic, and epigenetic factors [65,67].

This bidirectional communication network includes the CNS, the autonomic nervous system, and the enteric nervous system [68]. Moreover, multiple pathways seem to influence the microbiota-gut-brain axis, including (a) Neuroanatomic communication: occurs mainly via the vagus nerve [69,70]; (b) Hormones and neurotransmitters: the gastrointestinal tract produces hormones and neurotransmitters that bind to receptors on the vagus nerve, sending information to the brain. In addition, some hormones are also able to cross the BBB and act directly on the CNS [71,72]; (c) Neuroendocrine pathways: occur mainly via the HPA axis. The stress activates the HPA axis, culminating in the release of glucocorticoids such as cortisol from the adrenal cortex. Cortisol can change gastrointestinal motility, increase intestinal permeability, and affect the gut microbiota [73,74]. On the other hand, germ-free animals have exacerbated HPA stress response [75]; (d) Immunological pathways/inflammation: The gastrointestinal tract contains about 70–80% of the body’s immune cells [76]. Moreover, the intestinal microbiota also can influence the intestinal immune system and increase inflammatory cytokines (see below) [77,78]; (e) Bacteria-derived metabolites: especially short-chain fatty acids, which are produce by microbiota [79]; and (f) Neurotrophic factors: especially BDNF. The intestinal microbiota can modulate BDNF levels in the brain [80,81]. For example, a preclinical study showed that germ-free mice exhibited reduced BDNF expression levels in the cortex and hippocampus compared with specific pathogen-free mice [75]. 

It is worth highlighting that lipopolysaccharide (LPS), a cell wall component of Gram-negative bacteria, is recognized by the Toll-like receptors, which transduce signals for an increase in inflammatory cytokines [77,78]. Moreover, LPS receptors are also present in the afferent fibers of the vagus nerve; then, the activation of these receptors can send signals to the brain about what is happening in the gut [70,82]. Interestingly, the increased intestinal permeability, also called leaky gut, amplifies this response even more. Indeed, leaky gut increases the translocation of bacterial metabolic components, toxins, and food particles into the bloodstream and thus activates the immune response, contributing to systemic inflammation [83,84]. 

A good deal of data has established that the microbiota-gut-brain axis seems to influence several neuropsychiatric disorders [63,85,86]. Systematic reviews and meta-analyses evidenced that patients with irritable bowel syndrome or inflammatory bowel diseases had more depressive symptoms than healthy controls [87,88]. On the other hand, Maes et al. [89] revealed that individuals with MDD have more leaky gut and an increase in the LPS translocation. Interestingly, clinical studies [90,91,92] and a meta-analysis [62] have also shown that individuals with MDD present altered gut microbiota composition compared with controls. Additionally, meta-analytic studies of interventional clinical studies evidenced that probiotic treatment can reduce depressive symptoms [62,93]. 

The microbiota-gut-brain axis is also relevant to neurodegenerative diseases [94,95]. A meta-analysis demonstrated that people with constipation are at a higher risk of developing Parkinson’s disease [96]. Interestingly, irritable bowel syndrome [97] and inflammatory bowel disease [98] are associated with an increased risk of dementia. Moreover, data from the literature show that gut microbiota can contribute to the interindividual variability of clinical features of Parkinson’s disease [99]. For example, a study showed that decreases in Lachnospiraceae and increases in Lactobacillaceae and Christensenellaceae were associated with a worse clinical profile, including higher frequencies of cognitive impairment, gait disturbances, and postural instability [99]. Another clinical study revealed a decreased microbial diversity in Alzheimer’s diseases participants [100]. In line with this, a meta-analysis highlighted that probiotics improved cognitive performance and inflammation and oxidative stress biomarkers in Alzheimer’s disease and mild cognitive impairment individuals [101]. A clinical trial also showed that probiotics treatment improved constipation in Parkinson’s disease patients [102]. 

## 5. SARS-CoV-2 and Inflammation/Neuroinflammation

As already mentioned, individuals infected with SARS-CoV-2 can be asymptomatic or have severe symptoms. The factors that trigger the most severe form of the disease are not fully understood. It does not seem to be only related to viral load but also involves a defective interferon response [103]. Excessive inflammatory response to SARS-CoV-2 and the release of a cytokine storm mediated by membrane-bound immune receptors and downstream signaling pathways [104] are associated with disease severity and death in individuals with COVID-19 [105,106]. 

Some mechanisms to explain the hyperinflammatory state in the COVID-19 severity have been proposed. Release of IL-1β extimulated by pyroptotic death of infected cells along with danger-associated molecular patterns could contribute to inciting an inflammatory cascade [107]. Moreover, stem cell-derived type 2 alveolar cells infected with SARS-CoV-2 have been shown to upregulate expression of nuclear factor-kappa b (NFκB) target genes in vitro, including IL-6, CXCL8, CXCL2, CXCL3, CXCL10, and CXCL11 [108]. Individuals with severe COVID-19 have markedly reduced numbers of CD4+ and CD8+ T cells, and lymphocyte counts negatively correlate with levels of serum IL-6, IL-10, and TNF-α [109]. Some studies also showed that patients with severe COVID-19 exhibit higher levels of various proinflammatory cytokines, such as IL-2, IL-6, IL-7, IL-10, inducible protein 10, monocyte chemoattractant protein 1, TNF-α, macrophage inflammatory protein 1 alpha, and granulocyte-colony-stimulating factor [108,110,111]. Chen et al. [112] also reported that the serum SARS-CoV-2 viral load was closely associated with IL-6 levels in critical patients. It is worth noting that some meta-analyses have confirmed the influence of cytokines elevation, mainly IL-6, in severe COVID-19 [113,114].

As mentioned before, SARS-CoV-2 invades host cells via two receptors: ACE2 and CD147 [6,115]. Of note, the ACE2 receptor is expressed in regions of the human brain, including the motor cortex and posterior cingulate, nigra substance, ventricles, middle temporal gyrus, olfactory bulb, ventrolateral medulla, solitary tract nucleus, and vagus nerve. ACE2 is also expressed in neurons, microglia, astrocytes, and oligodendrocytes [116,117], suggesting the neuroinvasive capacity of SARS-CoV-2. After a SARS-CoV-2 spike protein interacts with ACE2, activation of the NLR family pyrin domain containing 3 (NLRP3) inflammasome will occur, and thus neuroinflammation [118]. Another study that investigated the expression level of ACE2 and CD147 receptors highlighted the relevance of CD147. Indeed, this study showed that CD147 is higher expressed in most brain cell lines and mouse brain tissues when comparing with lung cell lines and tissue [119]. It is worth mentioning that CD147 seems to regulate NFκB-mediated inflammatory response [120,121].

Notably, there are other mechanisms by which the virus can infect and cause damage to the CNS. One of these mechanisms is neural access, which involves transporting the virus through the nasal cavity and rhinopharynx through the olfactory and trigeminal nerves [122]. There is also the trojan horse mechanism, in which infected leukocytes serve as a dissemination vehicle and can cross the BBB [123]. Pro-inflammatory cytokines can disrupt the BBB and increase its permeability, allowing infected cells, cytokines, and even the virus to pass into the CNS [54,124,125]. In the CNS, inflammatory cytokines can induce neuroinflammation through microglial activation and proliferation [126,127,128]. In addition to microglia, astrocytes are also active players in neuroinflammation, and their response can be beneficial or detrimental for brain tissue repair, depending on timing and context [129]. 

In the presence of neuroinflammation, several brain changes can occur, including a reduction in neuroplasticity and neurogenesis, alteration in glutamate metabolism, changes in monoamine metabolism (decreasing serotonin and increasing kynurenine pathway), hyperactivation of HPA axis, increase in oxidative stress, neuronal dysfunction and death, increase in protein aggregates, and others factors that can be involved in that pathophysiology of neuropsychiatric and neurodegenerative diseases [25,30,130,131].

Allied to this context, several individuals infected by SARS-CoV-2 present neurological symptoms, including dizziness, headache, impaired consciousness, acute cerebrovascular disease, and ataxia [125,132]. In a study performed with 214 COVID-19 patients from Wuhan, China, 36.4% of patients had neurological symptoms, and they were more common in patients with severe infection (45.5%) [125]. A meta-analysis showed that the overall prevalence of depression, anxiety, and sleep disturbances among COVID-19 patients was 45%, 47%, and 34%, respectively. [133]. A longitudinal prospective study nested to a population cohort provided evidence of cognitive decline among individuals with mild symptomatic SARS-CoV-2 infection [16]. Researchers have also hypothesized that patients surviving COVID-19 may be at higher risk for subsequent development of neurological disease and, in particular cognitive decline and Alzheimer’s disease [134]. Additionally, a clinical study showed a worsening in motor and nonmotor symptoms in people with COVID-19 and Parkinson’s disease [135]. Although it is still uncertain, the increase in inflammatory cytokines may be responsible, at least in part, for the neuropsychiatric symptoms associated with COVID-19 [136].

## 6. SARS-CoV-2 and Microbiota-Gut-Brain Axis

In addition to the classic symptoms already mentioned, individuals with COVID-19 have more gastrointestinal symptoms, including nausea, vomiting, abdominal pain, and diarrhea [137,138,139,140]. Notably, a meta-analysis found that 17.6% of individuals with COVID-19 had gastrointestinal symptoms [141]. Moreover, it was shown that individuals with COVID-19 and gastrointestinal symptoms have a longer time from onset to admission and that gastrointestinal symptom usually became more pronounced with disease progression [142]. Interestingly, patients with diarrhea had higher stool RNA positivity and viral load than those without diarrhea [141]. Additionally, 70.3% of patients had prolonged shedding of viral RNA in the stool rather than respiratory samples [141]. On the other hand, a pilot study with 15 individuals with COVID-19 showed that 7 patients (without gastrointestinal symptoms) were detected positive for SARS-CoV-2 in the feces at baseline [143].

Interestingly, people with COVID-19 also have changes in the gut microbiota. A preliminary study highlighted that some patients with COVID-19 showed intestinal microbial dysbiosis with decreased probiotics such as *Lactobacillus* and *Bifidobacterium* [144]. In a pilot study of 15 patients with COVID-19 in Hong Kong, fecal samples were collected (two or three times per week from time of hospitalization until discharge) and microbial profiling of these fecal samples was performed with metagenomic sequencing. Patients with COVID-19 had significant alterations in fecal microbiota compared with controls, characterized by enrichment of opportunistic pathogens (including *Clostridium hathewayi*, *Actinomyces viscosus*, and *Bacteroides nordii*) and depletion of beneficial commensals at the time of hospitalization and at all time points during hospitalization [145]. Moreover, compared with individuals with COVID-19 who were not treated with antibiotics, individuals with COVID-19 who received antibiotics had greater depletion of symbiotic bacteria (including *Faecalibacterium prausnitzii*, *Lachnospiraceae bacterium 5_1_63FAA*, *Eubacterium rectale*, *Ruminococcus obeum*, and *Dorea formicigenerans*). Regardless of antibiotic use, dysbiosis persisted even after clearance of SARS-CoV-2 (determined from throat swabs) and resolution of respiratory symptoms [145]. Interestingly, the same study also showed that the baseline abundance of *Coprobacillus* (which upregulates colonic expression of ACE2 in murine gut [146]), *Clostridium ramosum*, and *Clostridium hathewayi* were positively correlated with COVID-19 severity. In contrast, *Alistipes onderdonkii* and *Faecalibacterium prausnitzii* (which has anti-inflammatory effects [147]) were negatively correlated with COVID-19 severity [145]. Over the course of hospitalization, *Bacteroides dorei*, *Bacteroides thetaiotaomicron*, *Bacteroides massiliensis*, and *Bacteroides ovatus* were inversely correlated with SARS-CoV-2 load in fecal samples from patients [145]. Noteworthy, these four species were associated with downregulation of ACE2 expression in the murine colon [146]. On the other hand, *Erysipelotrichaceae bacterium 2_2_44A* (implicated in gut inflammation [148]) was positively correlated with fecal SARS-CoV-2 load [145]. 

Another study conducted with a cohort of 84 patients (30 COVID-19 patients, 24 hospitalized patients with influenza A (H1N1) infection, and 30 healthy control) revealed that microbial diversity was decreased in COVID-19 and H1N1 patients when compared with healthy control. Interestingly, more than 50% of 1242 operational taxonomic units (OTUs) were shared by the three groups (COVID-19, H1N1, and control), and 62.3% of OTUs overlapped between the COVID-19 group and healthy control [149]. After analyzing bacterial taxonomic differences, it was demonstrated that, compared with healthy control, the abundance of the *Ruminococcaceae* family and several genera from the Lachnospiraceae family (*Fusicatenibacter*, *Anaerostipes*, *Agathobacter*, unclassified Lachnospiraceae, and *Eubacterium hallii group*) were reduced in COVID-19 patients. On the other hand, the number of *Streptococcus* (class Bacilli) was higher in COVID-19 individuals than in the other two groups [149]. Additionally, it was shown that bacterial taxonomic differences were present in the H1N1 group compared with the COVID-19 and control (see details in [149]).

The same study conducted by Gu et al. [149] also demonstrated differences in fecal microbiota. The gut microbiota of the COVID-19 group was dominated by *Streptococcus*, *Rothia*, *Veillonella*, *Erysipelatoclostridium*, and *Actinomyces*, whereas the microbiota of healthy control was dominated by the genera *Romboutsia*, *Faecalibacterium*, *Fusicatenibacter*, and *Eubacterium hallii* group [149]. Noteworthy, *Rhothia* spp. can cause several infections, including pneumonia, especially in immunocompromised individuals [150]. Another interesting result was that compared with healthy control, *Agathobacter*, *Fusicatenibacter*, *Roseburia,* and *Ruminococcaceae UCG−013* were depleted in COVID-19 patients and were negatively correlated mainly with CRP, procalcitonin, and D-dimer levels [149].

Another clinical study with 100 individuals with COVID-19 and 78 healthy controls also showed a change in the gut microbiota composition. At the phylum level, members of the *Bacteroidetes* were more relatively abundant in patients with COVID-19 compared with healthy individuals, whereas *Actinobacteria* were more relatively abundant in non-COVID-19 individuals. At the species level, without controlling the use of antibiotics, compositional differences in the gut microbiota of COVID-19 were primarily driven by enrichment of species including *Ruminococcus gnavus*, *Ruminococcus torques*, and *Bacteroides dorei* and depletion of *Bifidobacterium adolescentis*, *Faecalibacterium prausnitzii*, and *Eubacterium rectale*. When the analyzes considered the antibiotic effects, differences between cohorts were primarily linked to the enrichment of taxa such as *Parabacteroides*, *Sutterella wadsworthensis*, and *Bacteroides caccae* and depletion of *Adlercreutzia equolifaciens*, *Dorea formicigenerans*, and *Clostridium leptum* in COVID-19 individuals relative to healthy controls [151]. Noteworthy, *Faecalibacterium prausnitzii* and *Bifidobacterium bifidum* were negatively correlated with severity after adjusting for antibiotic use and age. The microbiota composition was also associated with elevated concentrations of inflammatory cytokines (including TNF-α and C-X-C motif ligand 10 (CXCL10)) and blood markers (including CRP, lactate dehydrogenase, aspartate aminotransferase, and gamma-glutamyl transferase) [151]. Interestingly, the gut microbiota of recovered patients (in samples collected up to 30 days after disease resolution) remained significantly distinct, enriched in species including *Bifidobacterium dentium* and *Lactobacillus ruminis* and depleted in *Eubacterium rectale*, *Ruminococcus bromii*, *Faecalibacterium prausnitzii*, and *Bifidobacterium longum* [151].

It is worth mentioning that fecal microbiota are associated with fecal viral activity of SARS-CoV-2. It was found that fecal samples with high SARS-CoV-2 infectivity had a higher abundance of the bacterial species *Collinsella aerofaciens*, *Collinsella tanakaei*, *Streptococcus infantis*, and *Morganella morganii*, compared with samples with low-to-none SARS-CoV-2 infectivity [143]. Notably, *Collinsella aerofaciens* and *Morganella morganii* have been associated with opportunistic human infections [152,153]. On the other hand, fecal samples with low-to-none SARS-CoV-2 infectivity had higher abundances of *Parabacteroides merdae*, *Bacteroides stercoris*, *Alistipes onderdonkii,* and *Lachnspiraceae bacterium* 1_1_57FAA, compared with those with high SARS-CoV-2 infectivity [143]. Interestingly, bacteria members from *Parabacteroides*, *Bacteroides,* and *Lachnospiraceae* are known producers of short-chain fatty acids [154,155].

A continually emerging body of evidence supports the role of the gut microbiota in communicating with several organs in the body (including adipose system, liver, brain, lung, and others) and influencing processes in health and disease [156]. Interestingly, lung tissue microbiota was evaluated in 20 fatal COVID-19 individuals. It was demonstrated that the most prevalent and regularly detected genera in all patients were *Acinetobacter* (80.70% of the total sequences), *Chryseobacterium* (2.68%), *Burkholderia* (2.00%), *Brevundimonas* (1.18%), *Sphingobium* (0.93%), and *Enterobacteriaceae* (0.68%), together comprising 92.32% of the total sequences. *Mycobacterium* (3.59%) and *Prevotella* (0.56%) were detected mainly in two patients. Moreover, the same study also evaluated the fungal community in the lung and revealed that the most common genus was *Cutaneotrichosporon* (*Cryptococcus*, 28.14%), followed by *Issatchenkia* (8.22%), *Wallemia* (4.77%), *Cladosporium* (4.67%), *Alternaria* (4.46%), *Dipodascus* (4.01%), *Mortierella* (3.22%), *Aspergillus* (2.72%), *Naganishia* (2.53%), *Diutina* (2.15%), and *Candida* (1.42%) [157]. It is worth noting that *Acinetobacter baumannii* (one of the species of *Acinetobacter)* is associated with resistance to various groups of antimicrobial agents [158]. The association between the nasal microbiota and viral infection has also received attention. Dysfunctional nasal microbiota can impair nasal mucosa, barrier integrity, and immune response, turning the viral infection into an overt disease [159].

One of the mechanisms of action that justifies these changes in the microbiota is related to ACE2. ACE2 is necessary for the surface expression of the neutral amino acid transporter B^0^AT1 (SLC6A19) in the small intestine. Dietary tryptophan is primarily absorbed via the B^0^AT1/ACE2 transport pathway in the small intestinal epithelial cells, and this results in the activation of the mammalian target of the rapamycin (mTOR) pathway, which regulates the expression of antimicrobial peptides. These antimicrobial peptides can affect the intestinal composition of the gut microbiota [160,161]. As previously mentioned, the SARS-CoV-2 S protein binds ACE2 and, when SARS-CoV-2 blocks ACE2, it also blocks B^0^AT1, and thus tryptophan cannot get absorbed efficiently, which leads to the aberrant secretion of antimicrobial peptides and consequently to an altered microbiota [162]. Noteworthy, these changes also confer susceptibility to inflammation of the large intestine [160,162]. 

It is worth noting that the severity of COVID-19 is associated with other pathologies, such as cardiovascular diseases (such as hypertension, diabetes, and cardia-cerebrovascular disease) [163,164] and chronic obstructive pulmonary disease [164], in addition to obesity [165]. Interestingly, these diseases alone are also associated with intestinal dysbiosis, contributing to the clinical condition of the patients [166,167,168,169].

Interestingly, researchers are suggesting the use of probiotics in people with COVID-19. Of note, this can be justified due to some properties of probiotics, including reducing inflammatory cytokines and gastrointestinal symptoms [170,171,172,173,174]. However, as far as we are aware, there are still no published clinical studies using probiotics in people with COVID-19, although there are ongoing phase II trials in the United Kingdom and the USA. Thus, we still cannot say whether the use of probiotics will bring any clinical benefit to these patients. On the other hand, treatments with probiotics have been shown to prevent respiratory tract infections in children and adults [175,176,177]. Of note, the guidance (version 5) established by China’s National Health Commission and National Administration of Traditional Chinese Medicine recommends the use of probiotics in patients with severe COVID-19 to preserve intestinal microbiota and to prevent secondary bacterial infections [178]. Moreover, as already mentioned, studies also evidenced that the use of probiotics improves depressive symptoms in healthy people with MDD [62,93], improves cognition in individuals with Alzheimer’s disease and mild cognitive impairment [101], and improves constipation in Parkinson’s disease patients [102].

Based on the above pieces of information, it is reasonable to assume that COVID-19 can affect the CNS and that microbiota and inflammation can, at least in part, be responsible for part of the effects of COVID-19 on the brain [65,179]. Notably, a clinical study with COVID-19 survivors evidenced that baseline systemic immune-inflammation index was positively associated with scores of depression and anxiety at one month follow-up after hospital treatment [180].

Finally, the decrease in tryptophan absorption induced by SARS-CoV-2 can also impact the brain because this amino acid is the precursor for the synthesis of serotonin, a neurotransmitter essential for mood regulation [181,182]. Moreover, changes in the microbiota per se also can influence the serotonergic neurotransmission in the gut and the CNS [181].

**Figure 1:**
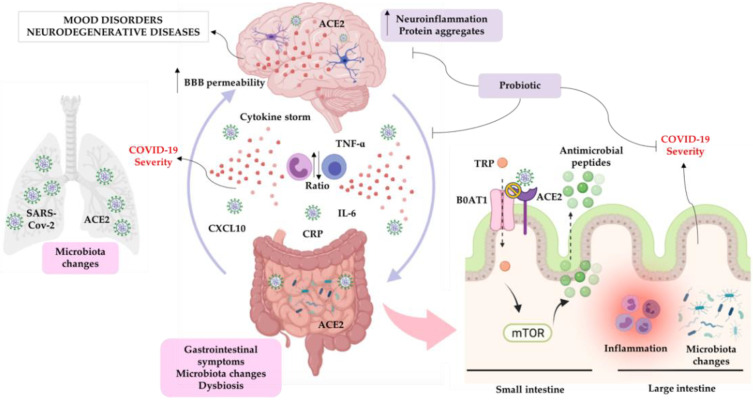
Possible mechanisms involved with microbiota changes and neuropsychiatric conditions in COVID-19. Individuals affected by SARS-Cov-2 have changes in lung microbiota, as well as changes in the gut microbiota, dysbiosis, and gastrointestinal symptoms. SARS-Cov-2 enters the respiratory system and systemic circulation but can also access other organs and systems, including gastrointestinal and central nervous systems. The main mechanism of entry of the virus into cells is mediated by the angiotensin-converting enzyme 2 (ACE-2) receptor, which is widely expressed in these systems. The virus itself is not the only factor associated with the COVID-19 severity; it is believed that elevated inflammatory markers, such as chemokine (C-X-C motif) ligand-10 (CXCL-10), interleukin-6 (IL-6), tumor necrosis factor-alpha (TNF-α), and C-reactive protein (CRP) and increased neutrophil-to-lymphocyte rate are also involved in disease severity. Systemic exacerbated inflammation could lead to an increase in blood–brain-barrier (BBB) permeability, leading to neuroinflammation. In addition, the virus can also activate microglia cells, leading to increased cytokines release and astrocyte activation, which are associated with the development and progression of mood disorders, including depression. These effects also can elevate protein aggregates that are involved in the pathophysiology of neurodegenerative diseases, such as Parkinson’s and Alzheimer’s diseases. In the small intestine, ACE-2 receptor is necessary for the surface expression of the neutral amino acid transporter B^0^AT1 (SLC6A19). Tryptophan (TRP) is mainly absorbed via the B^0^AT1/ACE2 transport pathway and induces the activation of the mammalian target of the rapamycin (mTOR) pathway, which regulates the expression of antimicrobial peptides, which are important to maintain an ideal microbiota in the large intestine. Thus, the block of this pathway by SARS-CoV-2 binding in the ACE-2 receptor could lead to inflammation and microbiota changes, which can be associated with the COVID-19 severity. There is bidirectional communication between brain and gut; thus, dysbiosis and exacerbated inflammation induced by COVID-19 could affect the brain and increase the risk of mood disorders and neurodegenerative diseases. Probiotic treatment could be an alternative treatment since it can reduce inflammation and improve gut microbiota. Images were extracted from Biorender app.

Figure 1 highlights the possible mechanisms involved with microbiota changes and neuropsychiatric conditions in COVID-19.

## 7. Future Directions and Conclusions

COVID-19 has had an impact all over the world. Although many studies have reported the pathophysiological aspects of the disease, it is still unclear how the virus affects some systems and organs. The severity of the disease appears to be related to an exacerbated immune system response that somehow could impact the CNS and justify the neurological and psychiatric symptoms that have been reported in many studies. Indeed, BBB disruption, microglial activation, and neuroinflammation have been involved in the development and progression of psychiatric and neurodegenerative diseases.

Although there are few studies, a relationship between the intestinal microbiota and COVID-19 has been reported. This correlation can be associated with the cytokine storm caused by the virus and its influence on antimicrobial peptides release, which is relevant to maintain healthy microbiota. Some COVID-19 patients have gastrointestinal symptoms, dysbiosis, and changes in the intestinal microbiota. These alterations can also be associated with inflammation and alteration in gut–brain axis communication. Future studies are suggested to evaluate the effectiveness of probiotics in reducing gastrointestinal symptoms and neuropsychiatric symptoms in patients with COVID-19.

## Data Availability

Not applicable.

## References

[1] WHO WHO Director-General’s Opening Remarks at the Media Briefing on COVID-19—11 March 2020. https://www.who.int/director-general/speeches/detail/who-director-general-s-opening-remarks-at-the-media-briefing-on-COVID-19---11-march-2020.

[2] WHO Weekly Epidemiological Update on COVID-19—15 June 2021. https://www.who.int/publications/m/item/weekly-epidemiological-update-on-covid-19---15-june-2021.

[3] Hoffmann M., Kleine-Weber H., Schroeder S., Krüger N., Herrler T., Erichsen S., Schiergens T.S., Herrler G., Wu N.H., Nitsche A. (2020). SARS-CoV-2 Cell Entry Depends on ACE2 and TMPRSS2 and Is Blocked by a Clinically Proven Protease Inhibitor. Cell.

[4] Gupta A., Madhavan M.V., Sehgal K., Nair N., Mahajan S., Sehrawat T.S., Bikdeli B., Ahluwalia N., Ausiello J.C., Wan E.Y. (2020). Extrapulmonary manifestations of COVID-19. Nat. Med..

[5] Wang K., Chen W., Zhou Y.-S., Lian J.-Q., Zhang Z., Du P., Gong L., Zhang Y., Cui H.-Y., Geng J.-J. (2020). SARS-CoV-2 invades host cells via a novel route: CD147-spike protein. bioRxiv.

[6] Wang K., Chen W., Zhang Z., Deng Y., Lian J.Q., Du P., Wei D., Zhang Y., Sun X.X., Gong L. (2020). CD147-spike protein is a novel route for SARS-CoV-2 infection to host cells. Signal. Transduct. Target..

[7] Ragab D., Salah Eldin H., Taeimah M., Khattab R., Salem R. (2020). The COVID-19 Cytokine Storm; What We Know So Far. Front. Immunol..

[8] Sinha P., Matthay M.A., Calfee C.S. (2020). Is a “cytokine Storm” Relevant to COVID-19?. JAMA Intern. Med..

[9] Mangalmurti N., Hunter C.A. (2020). Cytokine Storms: Understanding COVID-19. Immunity.

[10] Fernández-Rodríguez A., Casas I., Culebras E., Morilla E., Cohen M.C., Alberola J. (2020). COVID-19 and post-mortem microbiological studies. Span. J. Leg Med..

[11] Johnson K.D., Harris C., Cain J.K., Hummer C., Goyal H., Perisetti A. (2020). Pulmonary and Extra-Pulmonary Clinical Manifestations of COVID-19. Front. Med..

[12] Hartung H.P., Aktas O. (2020). COVID-19 and management of neuroimmunological disorders. Nat. Rev. Neurol..

[13] Pezzini A., Padovani A. (2020). Lifting the mask on neurological manifestations of COVID-19. Nat. Rev. Neurol..

[14] Torales J., O’Higgins M., Castaldelli-Maia J.M., Ventriglio A. (2020). The outbreak of COVID-19 coronavirus and its impact on global mental health. Int. J. Soc. Psychiatry.

[15] Krishnamoorthy Y., Nagarajan R., Saya G.K., Menon V. (2020). Prevalence of psychological morbidities among general population, healthcare workers and COVID-19 patients amidst the COVID-19 pandemic: A systematic review and meta-analysis. Psychiatry Res..

[16] Del Brutto O.H., Wu S., Mera R.M., Costa A.F., Recalde B.Y., Issa N.P. (2021). Cognitive decline among individuals with history of mild symptomatic SARS-CoV-2 infection: A longitudinal prospective study nested to a population cohort. Eur. J. Neurol..

[17] Fiani B., Covarrubias C., Desai A., Sekhon M., Jarrah R. (2020). A Contemporary Review of Neurological Sequelae of COVID-19. Front. Neurol..

[18] Bostanciklioglu M. (2020). Temporal correlation between neurological and gastrointestinal symptoms of sars-cov-2. Inflamm. Bowel Dis..

[19] Chaves Andrade M., Souza de Faria R., Avelino Mota Nobre S. (2020). COVID-19: Can the symptomatic SARS-CoV-2 infection affect the homeostasis of the gut-brain-microbiota axis?. Med. Hypotheses.

[20] Hyman S.E. (2008). A glimmer of light for neuropsychiatric disorders. Nature.

[21] WHO Depression. https://www.who.int/news-room/fact-sheets/detail/depression.

[22] Malhi G.S., Mann J.J. (2018). Depression. Lancet.

[23] Czarny P., Wigner P., Galecki P., Sliwinski T. (2018). The interplay between inflammation, oxidative stress, DNA damage, DNA repair and mitochondrial dysfunction in depression. Prog. Neuro-Psychopharmacol. Biol. Psychiatry.

[24] Levy M.J.F., Boulle F., Steinbusch H.W., van den Hove D.L.A., Kenis G., Lanfumey L. (2018). Neurotrophic factors and neuroplasticity pathways in the pathophysiology and treatment of depression. Psychopharmacology.

[25] Miller A.H., Maletic V., Raison C.L. (2009). Inflammation and Its Discontents: The Role of Cytokines in the Pathophysiology of Major Depression. Biol. Psychiatry.

[26] Lyketsos C.G., Carrillo M.C., Ryan J.M., Khachaturian A.S., Trzepacz P., Amatniek J., Cedarbaum J., Brashear R., Miller D.S. (2011). Neuropsychiatric symptoms in Alzheimer’s disease. Alzheimer’s Dement..

[27] Poewe W., Seppi K., Tanner C.M., Halliday G.M., Brundin P., Volkmann J., Schrag A.E., Lang A.E. (2017). Parkinson disease. Nat. Rev. Dis. Prim..

[28] Barnham K.J., Masters C.L., Bush A.I. (2004). Neurodegenerative diseases and oxidatives stress. Nat. Rev. Drug Discov..

[29] Soto C. (2003). Unfolding the role of protein misfolding in neurodegenerative diseases. Nat. Rev. Neurosci..

[30] Heneka M.T., Carson M.J., El Khoury J., Landreth G.E., Brosseron F., Feinstein D.L., Jacobs A.H., Wyss-Coray T., Vitorica J., Ransohoff R.M. (2015). Neuroinflammation in Alzheimer’s disease. Lancet Neurol..

[31] Hirsch E.C., Hunot S. (2009). Neuroinflammation in Parkinson’s disease: A target for neuroprotection?. Lancet Neurol..

[32] Lau A., Tymianski M. (2010). Glutamate receptors, neurotoxicity and neurodegeneration. Pflug. Arch. Eur. J. Physiol..

[33] Kumar A., Singh A., Ekavali (2015). A review on Alzheimer’s disease pathophysiology and its management: An update. Pharm. Rep..

[34] Furman D., Campisi J., Verdin E., Carrera-Bastos P., Targ S., Franceschi C., Ferrucci L., Gilroy D.W., Fasano A., Miller G.W. (2019). Chronic inflammation in the etiology of disease across the life span. Nat. Med..

[35] Nathan C., Ding A. (2010). Nonresolving Inflammation. Cell.

[36] Gisterå A., Hansson G.K. (2017). The immunology of atherosclerosis. Nat. Rev. Nephrol..

[37] Wellen K.E., Hotamisligil G.S. (2005). Inflammation, stress, and diabetes. J. Clin. Investig..

[38] Taniguchi K., Karin M. (2018). NF-B, inflammation, immunity and cancer: Coming of age. Nat. Rev. Immunol..

[39] Miller A.H., Raison C.L. (2016). The role of inflammation in depression: From evolutionary imperative to modern treatment target. Nat. Rev. Immunol..

[40] Perry V.H., Cunningham C., Holmes C. (2007). Systemic infections and inflammation affect chronic neurodegeneration. Nat. Rev. Immunol..

[41] Howren M.B., Lamkin D.M., Suls J. (2009). Associations of depression with c-reactive protein, IL-1, and IL-6: A meta-analysis. Psychosom Med..

[42] Dowlati Y., Herrmann N., Swardfager W., Liu H., Sham L., Reim E.K., Lanctôt K.L. (2010). A Meta-Analysis of Cytokines in Major Depression. Biol. Psychiatry.

[43] Smith K.J., Au B., Ollis L., Schmitz N. (2018). The association between C-reactive protein, Interleukin-6 and depression among older adults in the community: A systematic review and meta-analysis. Exp. Gerontol..

[44] Köhler C.A., Freitas T.H., Maes M., de Andrade N.Q., Liu C.S., Fernandes B.S., Stubbs B., Solmi M., Veronese N., Herrmann N. (2017). Peripheral cytokine and chemokine alterations in depression: A meta-analysis of 82 studies. Acta Psychiatr. Scand..

[45] Osimo E.F., Baxter L.J., Lewis G., Jones P.B., Khandaker G.M. (2019). Prevalence of low-grade inflammation in depression: A systematic review and meta-Analysis of CRP levels. Psychol. Med..

[46] Enache D., Pariante C.M., Mondelli V. (2019). Markers of central inflammation in major depressive disorder: A systematic review and meta-analysis of studies examining cerebrospinal fluid, positron emission tomography and post-mortem brain tissue. Brain Behav. Immun..

[47] Lai K.S.P., Liu C.S., Rau A., Lanctôt K.L., Köhler C.A., Pakosh M., Carvalho A.F., Herrmann N. (2017). Peripheral inflammatory markers in Alzheimer’s disease: A systematic review and meta-analysis of 175 studies. J. Neurol. Neurosurg. Psychiatry.

[48] Qin X.Y., Zhang S.P., Cao C., Loh Y.P., Cheng Y. (2016). Aberrations in peripheral inflammatory cytokine levels in Parkinson disease: A systematic review and meta-analysis. JAMA Neurol..

[49] Chen X., Hu Y., Cao Z., Liu Q., Cheng Y. (2018). Cerebrospinal fluid inflammatory cytokine aberrations in Alzheimer’s disease, Parkinson’s disease and amyotrophic lateral sclerosis: A systematic review and meta-analysis. Front. Immunol..

[50] Dantzer R., O’Connor J.C., Freund G.G., Johnson R.W., Kelley K.W. (2008). From inflammation to sickness and depression: When the immune system subjugates the brain. Nat. Rev. Neurosci..

[51] Kaufmann F.N., Costa A.P., Ghisleni G., Diaz A.P., Rodrigues A.L.S., Peluffo H., Kaster M.P. (2017). NLRP3 inflammasome-driven pathways in depression: Clinical and preclinical findings. Brain Behav. Immun..

[52] Haruwaka K., Ikegami A., Tachibana Y., Ohno N., Konishi H., Hashimoto A., Matsumoto M., Kato D., Ono R., Kiyama H. (2019). Dual microglia effects on blood brain barrier permeability induced by systemic inflammation. Nat. Commun..

[53] Kealy J., Greene C., Campbell M. (2020). Blood-brain barrier regulation in psychiatric disorders. Neurosci. Lett..

[54] Najjar S., Pearlman D.M., Devinsky O., Najjar A., Zagzag D. (2013). Neurovascular unit dysfunction with blood-brain barrier hyperpermeability contributes to major depressive disorder: A review of clinical and experimental evidence. J. Neuroinflamm..

[55] Sweeney M.D., Sagare A.P., Zlokovic B.V. (2018). Blood–brain barrier breakdown in Alzheimer disease and other neurodegenerative disorders. Nat. Rev. Neurol..

[56] Bowman G.L., Kaye J.A., Moore M., Waichunas D., Carlson N.E., Quinn J.F. (2007). Blood-brain barrier impairment in Alzheimer disease: Stability and functional significance. Neurology.

[57] Berk M., Williams L.J., Jacka F.N., O’Neil A., Pasco J.A., Moylan S., Allen N.B., Stuart A.L., Hayley A.C., Byrne M.L. (2013). So depression is an inflammatory disease, but where does the inflammation come from?. BMC Med..

[58] Gubert C., Kong G., Renoir T., Hannan A.J. (2020). Exercise, diet and stress as modulators of gut microbiota: Implications for neurodegenerative diseases. Neurobiol. Dis..

[59] Kamada N., Seo S.U., Chen G.Y., Núñez G. (2013). Role of the gut microbiota in immunity and inflammatory disease. Nat. Rev. Immunol..

[60] Maslowski K.M., Vieira A.T., Ng A., Kranich J., Sierro F., Di Y., Schilter H.C., Rolph M.S., MacKay F., Artis D. (2009). Regulation of inflammatory responses by gut microbiota and chemoattractant receptor GPR43. Nature.

[61] Morais L.H., Schreiber H.L., Mazmanian S.K. (2021). The gut microbiota–brain axis in behaviour and brain disorders. Nat. Rev. Microbiol..

[62] Sanada K., Nakajima S., Kurokawa S., Barceló-Soler A., Ikuse D., Hirata A., Yoshizawa A., Tomizawa Y., Salas-Valero M., Noda Y. (2020). Gut microbiota and majore depressive disorder: A systematic review and meta-analysis. J. Affect. Disord..

[63] Foster J.A., McVey Neufeld K.A. (2013). Gut-brain axis: How the microbiome influences anxiety and depression. Trends Neurosci..

[64] Mayer E.A. (2011). Gut feelings: The emerging biology of gut-brain communication. Nat. Rev. Neurosci..

[65] Cryan J.F., O’riordan K.J., Cowan C.S.M., Sandhu K.V., Bastiaanssen T.F.S., Boehme M., Codagnone M.G., Cussotto S., Fulling C., Golubeva A.V. (2019). The microbiota-gut-brain axis. Physiol. Rev..

[66] Sekirov I., Russell S.L., Antunes L.C.M., Finlay B.B. (2010). Gut microbiota in health and disease. Physiol. Rev..

[67] Stilling R.M., Dinan T.G., Cryan J.F. (2014). Microbial genes, brain & behaviour—Epigenetic regulation of the gut-brain axis. GenesBrain Behav..

[68] Carabotti M., Scirocco A., Maselli M.A., Severi C. (2015). The gut-brain axis: Interactions between enteric microbiota, central and enteric nervous systems. Ann. Gastroenterol..

[69] Rao M., Gershon M.D. (2016). The bowel and beyond: The enteric nervous system in neurological disorders. Nat. Rev. Gastroenterol. Hepatol..

[70] Breit S., Kupferberg A., Rogler G., Hasler G. (2018). Vagus nerve as modulator of the brain-gut axis in psychiatric and inflammatory disorders. Front. Psychiatry.

[71] Mittal R., Debs L.H., Patel A.P., Nguyen D., Patel K., O’Connor G., Grati M., Mittal J., Yan D., Eshraghi A.A. (2017). Neurotransmitters: The Critical Modulators Regulating Gut–Brain Axis. J. Cell Physiol..

[72] Strandwitz P. (2018). Neurotransmitter modulation by the gut microbiota. Brain Res..

[73] Konturek P.C., Brzozowski T., Konturek S.J. (2011). Stress and the gut: Pathophysiology, clinical consequences, diagnostic approach and treatment options. J. Physiol. Pharm..

[74] Vodička M., Ergang P., Hrnčíř T., Mikulecká A., Kvapilová P., Vagnerová K., Šestáková B., Fajstová A., Hermanová P., Hudcovic T. (2018). Microbiota affects the expression of genes involved in HPA axis regulation and local metabolism of glucocorticoids in chronic psychosocial stress. Brain Behav. Immun..

[75] Sudo N., Chida Y., Aiba Y., Sonoda J., Oyama N., Yu X.N., Kubo C., Koga Y. (2004). Postnatal microbial colonization programs the hypothalamic-pituitary-adrenal system for stress response in mice. J. Physiol..

[76] Yoo B.B., Mazmanian S.K. (2017). The Enteric Network: Interactions between the Immune and Nervous Systems of the Gut. Immunity.

[77] Powell N., Walker M.M., Talley N.J. (2017). The mucosal immune system: Master regulator of bidirectional gut-brain communications. Nat. Rev. Gastroenterol. Hepatol..

[78] Belkaid Y., Hand T.W. (2014). Role of the microbiota in immunity and inflammation. Cell.

[79] Dalile B., Van Oudenhove L., Vervliet B., Verbeke K. (2019). The role of short-chain fatty acids in microbiota-gut-brain communication. Nat. Rev. Gastroenterol. Hepatol..

[80] Maqsood R., Stone T.W. (2016). The Gut-Brain Axis, BDNF, NMDA and CNS Disorders. Neurochem. Res..

[81] Savignac H.M., Corona G., Mills H., Chen L., Spencer J.P.E., Tzortzis G., Burnet P.W.J. (2013). Prebiotic feeding elevates central brain derived neurotrophic factor, N-methyl-d-aspartate receptor subunits and d-serine. Neurochem. Int..

[82] Bonaz B., Bazin T., Pellissier S. (2018). The vagus nerve at the interface of the microbiota-gut-brain axis. Front. Neurosci..

[83] Obrenovich M. (2018). Leaky Gut, Leaky Brain?. Microorganisms.

[84] Camilleri M. (2019). Leaky gut: Mechanisms, measurement and clinical implications in humans. Gut.

[85] Kelly J.R., Keane V.O., Cryan J.F., Clarke G., Dinan T.G. (2019). Mood and Microbes: Gut to Brain Communication in Depression. Gastroenterol. Clin. North. Am..

[86] Manosso L.M., Lin J., Carlessi A.S., Recco K.C.C., Quevedo J., Gonçalves C.L., Réus G.Z. (2021). Sex-related patterns of the gut-microbiota-brain axis in the neuropsychiatric conditions. Brain Res. Bull..

[87] Fond G., Loundou A., Hamdani N., Boukouaci W., Dargel A., Oliveira J., Roger M., Tamouza R., Leboyer M., Boyer L. (2014). Anxiety and depression comorbidities in irritable bowel syndrome (IBS): A systematic review and meta-analysis. Eur. Arch. Psychiatry Clin. Neurosci..

[88] Mikocka-Walus A., Knowles S.R., Keefer L., Graff L. (2016). Controversies Revisited: A Systematic Review of the Comorbidity of Depression and Anxiety with Inflammatory Bowel Diseases. Inflamm. Bowel Dis..

[89] Maes M., Kubera M., Leunis J.-C. (2008). The gut-brain barrier in major depression: Intestinal mucosal dysfunction with an increased translocation of LPS from gram negative enterobacteria (leaky gut) plays a role in the inflammatory pathophysiology of depression. Neuroendocr. Lett..

[90] Kelly J.R., Borre Y., O’ Brien C., Patterson E., El Aidy S., Deane J., Kennedy P.J., Beers S., Scott K., Moloney G. (2016). Transferring the blues: Depression-associated gut microbiota induces neurobehavioural changes in the rat. J. Psychiatr. Res..

[91] Jiang H., Ling Z., Zhang Y., Mao H., Ma Z., Yin Y., Wang W., Tang W., Tan Z., Shi J. (2015). Altered fecal microbiota composition in patients with major depressive disorder. Brain Behav. Immun..

[92] Naseribafrouei A., Hestad K., Avershina E., Sekelja M., Linløkken A., Wilson R., Rudi K. (2014). Correlation between the human fecal microbiota and depression. Neurogastroenterol. Motil.

[93] Liu R.T., Walsh R.F.L., Sheehan A.E. (2019). Prebiotics and probiotics for depression and anxiety: A systematic review and meta-analysis of controlled clinical trials. Neurosci. Biobehav. Rev..

[94] Houser M.C., Tansey M.G. (2017). The gut-brain axis: Is intestinal inflammation a silent driver of Parkinson’s disease pathogenesis?. NPJ Park Dis..

[95] Westfall S., Lomis N., Kahouli I., Dia S.Y., Singh S.P., Prakash S. (2017). Microbiome, probiotics and neurodegenerative diseases: Deciphering the gut brain axis. Cell Mol. Life Sci..

[96] Adams-Carr K.L., Bestwick J.P., Shribman S., Lees A., Schrag A., Noyce A.J. (2016). Constipation preceding Parkinson’s disease: A systematic review and meta-analysis. J. Neurol. Neurosurg. Psychiatry.

[97] Chen C.H., Lin C.L., Kao C.H. (2016). Irritable bowel syndrome is associated with an increased risk of dementia: A nationwide population-based study. PLoS ONE.

[98] Zhang B., Wang H.E., Bai Y.M., Tsai S.J., Su T.P., Chen T.J., Wang Y.P., Chen M.H. (2021). Inflammatory bowel disease is associated with higher dementia risk: A nationwide longitudinal study. Gut.

[99] Barichella M., Severgnini M., Cilia R., Cassani E., Bolliri C., Caronni S., Ferri V., Cancello R., Ceccarani C., Faierman S. (2019). Unraveling gut microbiota in Parkinson’s disease and atypical parkinsonism. Mov. Disord..

[100] Vogt N.M., Kerby R.L., Dill-McFarland K.A., Harding S.J., Merluzzi A.P., Johnson S.C., Carlsson C.M., Asthana S., Zetterberg H., Blennow K. (2017). Gut microbiome alterations in Alzheimer’s disease. Sci. Rep..

[101] Deng H., Dong X., Chen M., Zou Z. (2020). Efficacy of probiotics on cognition, and biomarkers of inflammation and oxidative stress in adults with Alzheimer’s disease or mild cognitive impairment-A meta-analysis of randomized controlled trials. Aging.

[102] Tan A.H., Lim S.Y., Chong K.K., A Manap M.A.A., Hor J.W., Lim J.L., Low S.C., Chong C.W., Mahadeva S., Lang A.E. (2021). Probiotics for Constipation in Parkinson Disease: A Randomized Placebo-Controlled Study. Neurology.

[103] Hadjadj J., Yatim N., Barnabei L., Corneau A., Boussier J., Smith N., Péré H., Charbit B., Bondet V., Chenevier-Gobeaux C. (2020). Impaired type I interferon activity and inflammatory responses in severe COVID-19 patients. Science.

[104] Hussman J.P. (2020). Cellular and Molecular Pathways of COVID-19 and Potential Points of Therapeutic Intervention. Front. Pharm..

[105] Huang C., Wang Y., Li X., Ren L., Zhao J., Hu Y., Zhang L., Fan G., Xu J., Gu X. (2020). Clinical features of patients infected with 2019 novel coronavirus in Wuhan, China. Lancet.

[106] Mehta P., McAuley D.F., Brown M., Sanchez E., Tattersall R.S., Manson J.J. (2020). COVID-19: Consider cytokine storm syndromes and immunosuppression. Lancet.

[107] Tay M.Z., Poh C.M., Rénia L., MacAry P.A., Ng L.F.P. (2020). The trinity of COVID-19: Immunity, inflammation and intervention. Nat. Rev. Immunol..

[108] Huang J., Hume A.J., Abo K.M., Werder R.B., Villacorta-Martin C., Alysandratos K.D., Beermann M.L., Simone-Roach C., Lindstrom-Vautrin J., Olejnik J. (2020). SARS-CoV-2 Infection of Pluripotent Stem Cell-Derived Human Lung Alveolar Type 2 Cells Elicits a Rapid Epithelial-Intrinsic Inflammatory Response. Cell Stem Cell.

[109] Diao B., Wang C., Tan Y., Chen X., Liu Y., Ning L., Chen L., Li M., Liu Y., Wang G. (2020). Reduction and Functional Exhaustion of T Cells in Patients With Coronavirus Disease 2019 (COVID-19). Front. Immunol..

[110] Chen G., Wu D., Guo W., Cao Y., Huang D., Wang H., Wang T., Zhang X., Chen H., Yu H. (2020). Clinical and immunological features of severe and moderate coronavirus disease 2019. J. Clin. Investig..

[111] Liu J., Li S., Liu J., Liang B., Wang X., Wang H., Li W., Tong Q., Yi J., Zhao L. (2020). Longitudinal characteristics of lymphocyte responses and cytokine profiles in the peripheral blood of SARS-CoV-2 infected patients. EBioMedicine.

[112] Chen X., Zhao B., Qu Y., Chen Y., Xiong J., Feng Y., Men D., Huang Q., Liu Y., Yang B. (2020). Detectable Serum Severe Acute Respiratory Syndrome Coronavirus 2 Viral Load (RNAemia) Is Closely Correlated with Drastically Elevated Interleukin 6 Level in Critically Ill Patients with Coronavirus Disease 2019. Clin. Infect. Dis..

[113] Leisman D.E., Ronner L., Pinotti R., Taylor M.D., Sinha P., Calfee C.S., Hirayama A.V., Mastroiani F., Turtle C.J., Harhay M.O. (2020). Cytokine elevation in severe and critical COVID-19: A rapid systematic review, meta-analysis, and comparison with other inflammatory syndromes. Lancet Respir. Med..

[114] Henry B.M., De Oliveira M.H.S., Benoit S., Plebani M., Lippi G. (2020). Hematologic, biochemical and immune biomarker abnormalities associated with severe illness and mortality in coronavirus disease 2019 (COVID-19): A meta-analysis. Clin. Chem Lab. Med..

[115] Florindo H.F., Kleiner R., Vaskovich-Koubi D., Acúrcio R.C., Carreira B., Yeini E., Tiram G., Liubomirski Y., Satchi-Fainaro R. (2020). Immune-mediated approaches against COVID-19. Nat. Nanotechnol..

[116] Xia H., Lazartigues E. (2008). Angiotensin-converting enzyme 2 in the brain: Properties and future directions. J. Neurochem..

[117] Zubair A.S., McAlpine L.S., Gardin T., Farhadian S., Kuruvilla D.E., Spudich S. (2020). Neuropathogenesis and neurologic manifestations of the coronaviruses in the age of coronavirus disease 2019: A review. JAMA Neurol..

[118] Ribeiro D.E., Oliveira-Giacomelli Á., Glaser T., Arnaud-Sampaio V.F., Andrejew R., Dieckmann L., Baranova J., Lameu C., Ratajczak M.Z., Ulrich H. (2021). Hyperactivation of P2X7 receptors as a culprit of COVID-19 neuropathology. Mol. Psychiatry.

[119] Qiao J., Li W., Bao J., Peng Q., Wen D., Wang J., Sun B. (2020). The expression of SARS-CoV-2 receptor ACE2 and CD147, and protease TMPRSS2 in human and mouse brain cells and mouse brain tissues. Biochem. Biophys. Res. Commun..

[120] Huang Z., Meng S., Wang L., Wang Y., Chen T., Wang C. (2012). Suppression of oxLDL-induced MMP-9 and EMMPRIN expression by berberine via inhibition of NF-κB activation in human THP-1 macrophages. Anat. Rec..

[121] Yurchenko V., Constant S., Eisenmesser E., Bukrinsky M. (2010). Cyclophilin-CD147 interactions: A new target for anti-inflammatory therapeutics. Clin. Exp. Immunol..

[122] Li Y.C., Bai W.Z., Hashikawa T. (2020). The neuroinvasive potential of SARS-CoV2 may play a role in the respiratory failure of COVID-19 patients. J. Med. Virol..

[123] Desforges M., Le Coupanec A., Dubeau P., Bourgouin A., Lajoie L., Dubé M., Talbot P.J. (2019). Human coronaviruses and other respiratory viruses: Underestimated opportunistic pathogens of the central nervous system?. Viruses.

[124] Najjar S., Pahlajani S., De Sanctis V., Stern J.N.H., Najjar A., Chong D. (2017). Neurovascular Unit Dysfunction and Blood–Brain Barrier Hyperpermeability Contribute to Schizophrenia Neurobiology: A Theoretical Integration of Clinical and Experimental Evidence. Front. Psychiatry.

[125] Mao L., Jin H., Wang M., Hu Y., Chen S., He Q., Chang J., Hong C., Zhou Y., Wang D. (2020). Neurologic Manifestations of Hospitalized Patients with Coronavirus Disease 2019 in Wuhan, China. JAMA Neurol..

[126] John G.R., Lee S.C., Brosnan C.F. (2003). Cytokines: Powerful regulators of glial cell activation. Neuroscientist.

[127] Dantzer R. (2018). Neuroimmune interactions: From the brain to the immune system and vice versa. Physiol. Rev..

[128] Shigemoto-Mogami Y., Hoshikawa K., Sato K. (2018). Activated microglia disrupt the blood-brain barrier and induce chemokines and cytokines in a rat in vitro model. Front. Cell Neurosci..

[129] Colombo E., Farina C. (2016). Astrocytes: Key Regulators of Neuroinflammation. Trends Immunol..

[130] Glass C.K., Saijo K., Winner B., Marchetto M.C., Gage F.H. (2010). Mechanisms Underlying Inflammation in Neurodegeneration. Cell.

[131] Leng F., Edison P. (2021). Neuroinflammation and microglial activation in Alzheimer disease: Where do we go from here?. Nat. Rev. Neurol..

[132] Tsivgoulis G., Palaiodimou L., Katsanos A.H., Caso V., Köhrmann M., Molina C., Cordonnier C., Fischer U., Kelly P., Sharma V.K. (2020). Neurological manifestations and implications of COVID-19 pandemic. Adv. Neurol. Disord..

[133] Deng J., Zhou F., Hou W., Silver Z., Wong C.Y., Chang O., Huang E., Zuo Q.K. (2021). The prevalence of depression, anxiety, and sleep disturbances in COVID-19 patients: A meta-analysis. Ann. N. Y. Acad Sci..

[134] Heneka M.T., Golenbock D., Latz E., Morgan D., Brown R. (2020). Immediate and long-term consequences of COVID-19 infections for the development of neurological disease. Alzheimer’s Res..

[135] Cilia R., Bonvegna S., Straccia G., Andreasi N.G., Elia A.E., Romito L.M., Devigili G., Cereda E., Eleopra R. (2020). Effects of COVID-19 on Parkinson’s Disease Clinical Features: A Community-Based Case-Control Study. Mov. Disord..

[136] Kappelmann N., Dantzer R., Khandaker G.M. (2021). Interleukin-6 as potential mediator of long-term neuropsychiatric symptoms of COVID-19. Psychoneuroendocrinology.

[137] Jin X., Lian J.S., Hu J.H., Gao J., Zheng L., Zhang Y.M., Hao S.R., Jia H.Y., Cai H., Zhang X.L. (2020). Epidemiological, clinical and virological characteristics of 74 cases of coronavirus-infected disease 2019 (COVID-19) with gastrointestinal symptoms. Gut.

[138] Galanopoulos M., Gkeros F., Doukatas A., Karianakis G., Pontas C., Tsoukalas N., Viazis N., Liatsos C., Mantzaris G.J. (2020). COVID-19 pandemic: Pathophysiology and manifestations from the gastrointestinal tract. World J. Gastroenterol..

[139] Wang D., Hu B., Hu C., Zhu F., Liu X., Zhang J., Wang B., Xiang H., Cheng Z., Xiong Y. (2020). Clinical Characteristics of 138 Hospitalized Patients with 2019 Novel Coronavirus-Infected Pneumonia in Wuhan, China. JAMA.

[140] Guan W., Ni Z., Hu Y., Liang W., Ou C., He J., Liu L., Shan H., Lei C., Hui D.S.C. (2020). Clinical Characteristics of Coronavirus Disease 2019 in China. N. Engl J. Med..

[141] Cheung K.S., Hung I.F.N., Chan P.P.Y., Lung K.C., Tso E., Liu R., Ng Y.Y., Chu M.Y., Chung T.W.H., Tam A.R. (2020). Gastrointestinal Manifestations of SARS-CoV-2 Infection and Virus Load in Fecal Samples from a Hong Kong Cohort: Systematic Review and Meta-analysis. Gastroenterology.

[142] Pan L., Mu M., Yang P., Sun Y., Wang R., Yan J., Li P., Hu B., Wang J., Hu C. (2020). Clinical characteristics of COVID-19 patients with digestive symptoms in Hubei, China: A descriptive, cross-sectional, multicenter study. Am. J. Gastroenterol..

[143] Zuo T., Liu Q., Zhang F., Lui G.C.Y., Tso E.Y.K., Yeoh Y.K., Chen Z., Boon S.S., Chan F.K.L., Chan P.K.S. (2021). Depicting SARS-CoV-2 faecal viral activity in association with gut microbiota composition in patients with COVID-19. Gut.

[144] Xu K., Cai H., Shen Y., Ni Q., Chen Y., Hu S., Li J., Wang H., Yu L., Huang H. (2020). Management of COVID-19: The Zhejiang experience. Zhejiang Da Xue Xue Bao Yi Xue Ban.

[145] Zuo T., Zhang F., Lui G.C.Y., Yeoh Y.K., Li A.Y.L., Zhan H., Wan Y., Chung A.C.K., Cheung C.P., Chen N. (2020). Alterations in Gut Microbiota of Patients With COVID-19 During Time of Hospitalization. Gastroenterology.

[146] Geva-Zatorsky N., Sefik E., Kua L., Pasman L., Tan T.G., Ortiz-Lopez A., Yanortsang T.B., Yang L., Jupp R., Mathis D. (2017). Mining the Human Gut Microbiota for Immunomodulatory Organisms. Cell.

[147] Miquel S., Martín R., Rossi O., Bermúdez-Humarán L.G., Chatel J.M., Sokol H., Thomas M., Wells J.M., Langella P. (2013). Faecalibacterium prausnitzii and human intestinal health. Curr. Opin. Microbiol..

[148] Kaakoush N.O. (2015). Insights into the role of Erysipelotrichaceae in the human host. Front. Cell Infect. Microbiol..

[149] Gu S., Chen Y., Wu Z., Chen Y., Gao H., Lv L., Guo F., Zhang X., Luo R., Huang C. (2020). Alterations of the gut microbiota in patients with coronavirus disease 2019 or H1N1 influenza. Clin. Infect. Dis..

[150] Ramanan P., Barreto J.N., Osmon D.R., Tosh P.K. (2014). Rothia bacteremia: A 10-year experience at Mayo Clinic, Rochester, Minnesota. J. Clin. Microbiol..

[151] Yeoh Y.K., Zuo T., Lui G.C.Y., Zhang F., Liu Q., Li A.Y.L., Chung A.C.K., Cheung C.P., Tso E.Y.K., Fung K.S.C. (2021). Gut microbiota composition reflects disease severity and dysfunctional immune responses in patients with COVID-19. Gut.

[152] Liu H., Zhu J., Hu Q., Rao X. (2016). Morganella morganii, a non-negligent opportunistic pathogen. Int. J. Infect. Dis..

[153] Shinagawa N., Taniguchi M., Hirata K., Furuhata T., Fukuhara K., Mizugucwi T., Osanai H., Yanai Y., Hata F., Kihara C. (2014). Bacteria isolated from surgical infections and its susceptibilities to antimicrobial agents—Special references to bacteria isolated between April 2010 and March 2011. Jpn J. Antibiot..

[154] Hwang N., Eom T., Gupta S.K., Jeong S.Y., Jeong D.Y., Kim Y.S., Lee J.H., Sadowsky M.J., Unno T. (2017). Genes and gut bacteria involved in luminal butyrate reduction caused by diet and loperamide. Genes.

[155] Kläring K., Just S., Lagkouvardos I., Hanske L., Haller D., Blaut M., Wenning M., Clavel T. (2015). Murimonas intestini gen. nov., sp. nov., an acetateproducing bacterium of the family Lachnospiraceae isolated from the mouse gut. Int. J. Syst Evol. Microbiol..

[156] Schroeder B.O., Bäckhed F. (2016). Signals from the gut microbiota to distant organs in physiology and disease. Nat. Med..

[157] Fan J., Li X., Gao Y., Zhou J., Wang S., Huang B., Wu J., Cao Q., Chen Y., Wang Z. (2020). The lung tissue microbiota features of 20 deceased patients with COVID-19. J. Clean Prod..

[158] Doi Y., Murray G.L., Peleg A.Y. (2015). Acinetobacter baumannii: Evolution of antimicrobial resistance-treatment options. Semin. Respir. Crit. Care Med..

[159] Di Stadio A., Costantini C., Renga G., Pariano M., Ricci G., Romani L. (2020). The microbiota/host immune system interaction in the nose to protect from COVID-19. Life.

[160] Hashimoto T., Perlot T., Rehman A., Trichereau J., Ishiguro H., Paolino M., Sigl V., Hanada T., Hanada R., Lipinski S. (2012). ACE2 links amino acid malnutrition to microbial ecology and intestinal inflammation. Nature.

[161] Perlot T., Penninger J.M. (2013). ACE2—From the renin-angiotensin system to gut microbiota and malnutrition. Microbes Infect..

[162] Mönkemüller K., Fry L.C., Rickes S. (2020). Covid-19, Coronavirus, SARS-CoV-2 and the small bowel. Rev. Esp. Enferm. Dig..

[163] Li B., Yang J., Zhao F., Zhi L., Wang X., Liu L., Bi Z., Zhao Y. (2020). Prevalence and impact of cardiovascular metabolic diseases on COVID-19 in China. Clin. Res. Cardiol..

[164] Wang B., Li R., Lu Z., Huang Y. (2020). Does comorbidity increase the risk of patients with covid-19: Evidence from meta-analysis. Aging.

[165] Demeulemeester F., de Punder K., van Heijningen M., van Doesburg F. (2021). Obesity as a Risk Factor for Severe COVID-19 and Complications: A Review. Cells.

[166] Musso G., Gambino R., Cassader M. (2010). Obesity, diabetes, and gut microbiota: The hygiene hypothesis expanded?. Diabetes Care.

[167] Ortega M.A., Fraile-Martínez O., Naya I., García-Honduvilla N., Álvarez-Mon M., Buján J., Asúnsolo Á., de la Torre B. (2020). Type 2 diabetes mellitus associated with obesity (Diabesity). The central role of gut microbiota and its translational applications. Nutrients.

[168] Tang W.H.W., Kitai T., Hazen S.L. (2017). Gut microbiota in cardiovascular health and disease. Circ. Res..

[169] Kazemian N., Mahmoudi M., Halperin F., Wu J.C., Pakpour S. (2020). Gut microbiota and cardiovascular disease: Opportunities and challenges. Microbiome.

[170] Mak J.W.Y., Chan F.K.L., Ng S.C. (2020). Probiotics and COVID-19: One size does not fit all. Lancet Gastroenterol. Hepatol..

[171] Akour A. (2020). Probiotics and COVID-19: Is there any link?. Lett. Appl. Microbiol..

[172] Bottari B., Castellone V., Neviani E. (2021). Probiotics and Covid-19. Int. J. Food Sci. Nutr..

[173] Sundararaman A., Ray M., Ravindra P.V., Halami P.M. (2020). Role of probiotics to combat viral infections with emphasis on COVID-19. Appl. Microbiol. Biotechnol..

[174] Mahooti M., Miri S.M., Abdolalipour E., Ghaemi A. (2020). The immunomodulatory effects of probiotics on respiratory viral infections: A hint for COVID-19 treatment?. Microb. Pathog..

[175] Hao Q., Dong B.R., Wu T. (2015). Probiotics for preventing acute upper respiratory tract infections. Cochrane Database Syst. Rev..

[176] West N.P., Horn P.L., Pyne D.B., Gebski V.J., Lahtinen S.J., Fricker P.A., Cripps A.W. (2014). Probiotic supplementation for respiratory and gastrointestinal illness symptoms in healthy physically active individuals. Clin. Nutr..

[177] Wang Y., Li X., Ge T., Xiao Y., Liao Y., Cui Y., Zhang Y., Ho W., Yu G., Zhang T. (2016). Probiotics for prevention and treatment of respiratory tract infections in children: A systematic review and meta-analysis of randomized controlled trials. Medicine.

[178] Gao Q.Y., Chen Y.X., Fang J.Y. (2020). 2019 Novel coronavirus infection and gastrointestinal tract. J. Dig. Dis..

[179] Serra D., Almeida L.M., Dinis T.C.P. (2019). The Impact of Chronic Intestinal Inflammation on Brain Disorders: The Microbiota-Gut-Brain Axis. Mol. Neurobiol..

[180] Mazza M.G., De Lorenzo R., Conte C., Poletti S., Vai B., Bollettini I., Melloni E.M.T., Furlan R., Ciceri F., Rovere-Querini P. (2020). Anxiety and depression in COVID-19 survivors: Role of inflammatory and clinical predictors. Brain Behav. Immun..

[181] O’Mahony S.M., Clarke G., Borre Y.E., Dinan T.G., Cryan J.F. (2015). Serotonin, tryptophan metabolism and the brain-gut-microbiome axis. Behav. Brain Res..

[182] Fernstrom J.D., Wurtman R.J. (1971). Brain serotonin content: Physiological dependence on plasma tryptophan levels. Science.

